# The role of TSPO PET in assessing neuroinflammation

**DOI:** 10.1007/s00415-017-8565-1

**Published:** 2017-07-11

**Authors:** Ania A. Crawshaw, Neil P. Robertson

**Affiliations:** Division of Psychological Medicine and Clinical Neuroscience, Department of Neurology, University Hospital of Wales, Cardiff University, Heath Park, Cardiff, CF14 4XN UK

## Introduction

Neuroinflammation is associated with increased expression of the 18-kDa translocator protein (TSPO), also known as the peripheral benzodiazepine receptor (PBR), which is present on the mitochondria of activated microglia, astroglia and macrophages. TSPO positron emission tomography (PET) ligands have been used in neuroinflammation research for over 20 years. The first-generation tracer ^11^C-PK11195 has some limitations including low signal-to-noise ratio making more subtle neuroinflammation difficult to detect in brain tissue. More recently, second- and third-generation TSPO-specific radiotracers have been developed to overcome these hurdles and have proven useful in assessment of neuroinflammation in a variety of neurological and psychiatric conditions. However, one drawback of the newer ligands is the need to genotype for rs6971 polymorphisms prior to imaging. The polymorphism determines radiotracer uptake since low-affinity binders (Thr/Thr) have to be excluded, and statistical analyses require adjustments depending on whether participants are mixed (Ala/Thr) or high affinity (Ala/Ala) binders.

This month’s journal club examines four papers utilising TSPO PET imaging in HIV, traumatic brain injury, multiple sclerosis (MS) and HTLV-1-associated myelopathy (HAM), respectively. This imaging modality is used variously in these four studies to explore questions regarding disease pathogenesis, response to treatment, and clinicopathological correlates.

## Neuroinflammation in treated HIV-positive individuals: a TSPO PET study

Despite the advent of combined antiretroviral therapy and the consequent effective control of HIV RNA in both the serum and CSF, many HIV-positive patients still develop HIV-associated brain disease, including cognitive impairment. It has been suggested that inflammation continues in the brain, despite undetectable viral loads, and that this inflammation is responsible for the gradually accruing cognitive deficits evident in many treated HIV patients.

One of the hypotheses regarding the mechanism of chronic immune activation in treated HIV patients relates to the concept of microbial translocation: the passage of gut flora into the bloodstream due to early CD4 T cell depletion in the gastrointestinal tract in the first few weeks of HIV infection, causing a breach in the mucosal immune system. The consequent presence of microbes in the systemic circulation (without overt bacteraemia) is postulated to be causative in the subsequent chronic inflammatory state in treated HIV patients.

This cross-sectional, case–control study explored the presence of microglial activation in cognitively healthy HIV-positive patients on suppressive antiretroviral therapy, correlating this with brain structure and function, peripheral chemokines and markers of microbial translocation. Twelve treated HIV patients and ten controls underwent [^11^C]PBR28 PET CT scans, volumetric and diffusion MR imaging, cognitive testing, measurement of CSF chemokines and HIV RNA, as well as PCR of plasma ribosomal 16s rRNA (a marker of microbial translocation).

Microglial activation, as measured by TSPO PET, was globally higher in treated HIV patients compared to controls. TSPO uptake was greatest in the subcortical grey matter, the basal ganglia in particular. Increased TSPO binding correlated with poorer performance in verbal learning and memory domains on cognitive testing. Increased TSPO binding and white matter mean diffusivity (MD) on diffusion imaging also correlated positively. Markers of microbial translocation correlated with increased TSPO binding, leading the authors to postulate that this phenomenon may be a causative factor in brain inflammation in treated HIV positive patients.

*Comment* This study lends weight to the hypothesis that chronic immune activation continues in HIV patients despite apparently effective antiretroviral treatment and that the consequent inflammation may be responsible for HIV-associated neurocognitive impairment. Nevertheless, these patients were by definition asymptomatic, so any relatively poorer performance on cognitive testing was, therefore, subclinical. A longitudinal study might be better placed to tackle the issue of causality.

Although this paper contributes to our understanding of pathophysiology in treated HIV patients, a study comparing TSPO uptake in asymptomatic HIV patients and those with HIV-associated cognitive impairment would be more likely to determine whether this imaging modality will prove clinically useful in this arena.

Vera J et al (2016) Neurology 86(15):1425–1432.

## Imaging of glial cell activation and white matter integrity in brains of active and recently retired National Football League players

In traumatic brain injury, it is thought that single and repeated insults can lead to prolonged immune activation. This study aimed to explore whether microglial activation is ongoing in the years following sports-related concussive and sub-concussive injuries. Ten young former and four active National Football League (NFL) players were recruited, in parallel with 16 controls, to undergo [^11^C]DPA-713 TSPO imaging alongside structural and diffusion MRI and neuropsychological testing. Mean time since last concussion was 7 years (range 1–21).

TSPO imaging revealed increased microglial activation in the NFL players in 8 of 12 regions measured, including the supramarginal gyri, mesial temporal lobes and left temporal pole. There was also reduced fractional anisotropy on DTI in six of ten regions measured, reflective of likely altered white matter integrity. There were no volumetric differences on structural MR, nor were there any differences in neuropsychological parameters.

*Comment* Although none of the players in this study have developed cognitive impairment at this stage in their lives, there is some evidence that collision sport professionals are more likely to develop cognitive, affective and behavioural changes later in life. Microglial activation as measured with TSPO PET may be one possible mechanism of this; however, a longitudinal study might be better placed to answer this question.

The authors hypothesise that microglial activation pathways may be therapeutic targets for preventative interventions before the clinical effects of recurrent head injury manifest. Given that microglia have reparative functions as well as deleterious, this is unlikely to be a straightforward undertaking. Another confounding factor when considering therapeutic targets is the poor specificity of TSPO radioligands for microglia since they also bind activated astroglia and macrophages.

Coughlin JM et al (2017) JAMA Neurol 74(1):67–74.

## Evaluation of the effect of fingolimod treatment on microglial activation using serial PET imaging in multiple sclerosis

TSPO PET has been used extensively in MS research. As might be expected, radiotracer uptake is increased in regions that correspond to T2 lesions on MRI. Interestingly, there is also increased tracer uptake in the normal appearing white matter (NAWM) and grey matter (GM), both in relapsing remitting MS and in progressive disease compared with healthy controls.

This study used ^11^C-(R)-PK11195 PET scans and MRI to evaluate the effect of fingolimod on neuroinflammation in patients with relapsing remitting MS switching from another disease-modifying treatment. Ten patients underwent scans at baseline and 6 months. Seven of these also had intermediate scans at 2 months. Eight controls were imaged for comparison.

Baseline scans, as expected from previous studies, revealed higher TSPO binding in the T2 lesions, NAWM and grey matter compared with controls. After 6 months on fingolimod, TSPO binding was 12.31% lower (*p* = 0.040) in the T2 lesions compared with baseline, but not in the NAWM or GM. Interestingly, TSPO binding in the NAWM and GM at 2 months was slightly (but not significantly) higher in the majority of patients compared to baseline.

*Comment* MRI has limited utility in monitoring response to treatment in MS, so this is one area where TSPO imaging could soon change clinical practice, both in the management of individual patients and in the development of novel disease-modifying treatments, whether for relapsing remitting or progressive disease. Despite the unfavourable signal-to-noise ratio with ^11^C-(R)-PK11195, significant differences were still seen in TSPO binding. Unlike novel TSPO radioligands, genetic polymorphisms do not affect binding affinity of ^11^C-(R)-PK11195—an important practical consideration if TSPO imaging is to be useful in clinical practice.

Suckersdorff M et al (2017) J Nucl Med ppjnumed.116.183020-32.

## Evidence of brain inflammation in patients with human T-lymphotropic virus type 1-associated myelopathy (HAM): a multimodal imaging study using ^11^C-PBR28 PET, MR T1-weighted and diffusion-weighted imaging

More than 10 million people worldwide are infected with HTLV-1 of whom 2–4% develop HAM. Clinically, the myelopathy manifests predominantly in the thoracic cord; however, MRI and histological studies have revealed that brain inflammation is often present too, albeit subclinically. The pathogenesis is thought to be a bystander effect of the cellular immune response to HTLV-1-infected lymphocytes that enter the central nervous system. At present the only available treatments have limited efficacy.

This pilot study examined the brains of five HAM patients and two asymptomatic HTLV-1 carriers. Historic healthy control data were also available for analysis. All participants underwent [^11^C]PBR28 PET, MRI (T1-weighted and diffusion sequences) and clinical assessment (including cognitive testing and objective gait measures). There were no consistent abnormalities on neuropsychology. All participants were identified as high-affinity binders on TSPO genotyping.

Global TSPO binding was significantly higher in HAM patients compared to asymptomatic carriers. Binding was also regionally increased in the thalamus. The two HAM subjects with severe disease had highest tracer uptake, followed by those with moderate and mild disease in decreasing order. Thalamic and brainstem GM volumes were reduced in HAM compared to healthy controls and correlated with disease severity, while thalamic mean diffusivity was increased in HAM-severe patients compared with controls.

*Comment* Despite such a small number of study participants, the differences demonstrated here are significant. The correlation of brain inflammation in HAM with measures of clinical severity suggests that TSPO PET could play an important role in future therapeutic research in HTLV. It might even prove useful in monitoring individual disease activity, though genetic polymorphisms determining affinity binding may prove to be significant hurdles. Research of TSPO ligand uptake in the cord may also be worth pursuing.

Dimber R et al (2016) J Nucl Med 15(12):1905–1912.

